# Understanding force-generating microtubule systems through *in vitro* reconstitution

**DOI:** 10.1080/19336918.2016.1241923

**Published:** 2016-10-07

**Authors:** Mathijs Vleugel, Maurits Kok, Marileen Dogterom

**Affiliations:** Department of Bionanoscience, Kavli Institute of Nanoscience, Faculty of Applied Sciences, Delft Institute of Technology, Delft, The Netherlands

**Keywords:** dynamic instability, *in vitro* reconstitution, MAPs, microtubules, pulling forces, pushing forces

## Abstract

Microtubules switch between growing and shrinking states, a feature known as dynamic instability. The biochemical parameters underlying dynamic instability are modulated by a wide variety of microtubule-associated proteins that enable the strict control of microtubule dynamics in cells. The forces generated by controlled growth and shrinkage of microtubules drive a large range of processes, including organelle positioning, mitotic spindle assembly, and chromosome segregation. In the past decade, our understanding of microtubule dynamics and microtubule force generation has progressed significantly. Here, we review the microtubule-intrinsic process of dynamic instability, the effect of external factors on this process, and how the resulting forces act on various biological systems. Recently, reconstitution-based approaches have strongly benefited from extensive biochemical and biophysical characterization of individual components that are involved in regulating or transmitting microtubule-driven forces. We will focus on the current state of reconstituting increasingly complex biological systems and provide new directions for future developments.

## General introduction

Together with actin and intermediate filaments, microtubules constitute the cytoskeleton of eukaryotic cells. In contrast to what is implied by the term ‘cytoskeleton’ (literally: skeleton of the cell), microtubules are generally very dynamic, and support a broad range of functions within the cell. For example, microtubules are essential for cell mechanics, cell division, intracellular transport, and cell motility. Whereas the minus-ends of microtubules are usually stabilised by other structures, the plus-ends constantly switch between growing and shrinking states. The energy released by microtubule growth as well as shrinkage is used for force generation in a wide variety of cellular processes. Over the past 2 decades, significant advancements have been made in our basic understanding of the diverse range of biological functions that are supported by microtubule-generated forces. The recent development of more sophisticated reconstitution systems allows for a more in-depth characterization of the biochemical and biophysical processes underlying microtubule dynamics and force generation.

In this review, we summarize our current understanding of the biochemical properties that regulate microtubule growth and shrinkage (see “Introduction to microtubule dynamics”) and describe how microtubule-associated proteins (MAPs) and other factors affect these parameters both *in vivo* and *in vitro* (see “Modulating microtubule dynamics”). In addition, we will discuss the biophysical principles behind the generation of pushing and pulling forces by microtubule-dynamics (see “Biophysical principles behind microtubule pushing forces” and “Biophysical principles behind microtubule pulling forces”) and provide examples of biological processes that rely on these forces (see “Pushing forces generated by microtubule polymerization” and “Pulling forces generated by microtubule depolymerization”). Finally, we highlight recent advancements that have been made by studying force-generation by microtubules in increasingly complex synthetic biology-based systems, ranging from single-molecule approaches to 3D-reconstitution assays (see “Reconstituting microtubule pushing forces”, “Reconstituting microtubule pulling forces,” and “Reconstituting complex force-generating microtubule systems”).

## Introduction to microtubule dynamics

**Figure 1.**
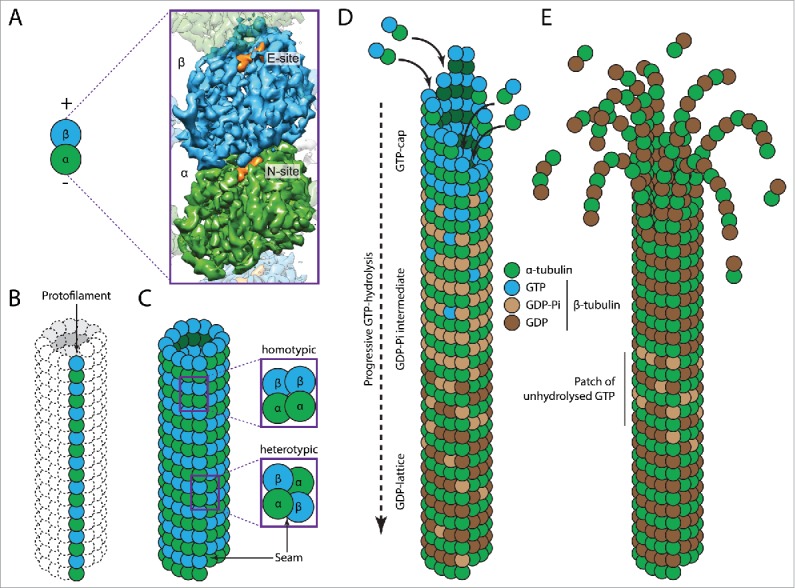
Biochemical basis of microtubule dynamics. (A) Schematic representation (left) and high-resolution cryo-EM structure (right),^6^ of α- (green) and β- (blue) tubulin dimers, showing the non-exchangeable (N-site) and exchangeable (E-site) nucleotide-binding sites. (B) Head-to-tail assembly of tubulin dimers into a single protofilament. (C) Assembly of 13 protofilaments into a cylindrical microtubule. The enlargements show homotypic (α-α and β-β) lateral contacts between the protofilaments and heterotypic (α-β and β-α) lateral contacts between protofilaments at the seam. (D) Growing microtubules incorporate GTP-bound α/β-tubulin dimers, resulting in a GTP-rich cap. Tubulin incorporation promotes the progressive hydrolysis of the β-tubulin bound GTP (blue) molecule into GDP (brown) via a GDP-Pi (beige) intermediate. (E) Microtubule undergoing catastrophe with protofilaments bending outwards. The GDP-lattice contains regions that are enriched in GTP-bound β-tubulin that can promote rescue events.

Microtubules are hollow, cylindrical polymers and are composed of heterodimers of α- and β–tubulin subunits that assemble in a head-to-tail fashion (Fig. 1A). Linear arrays of α/β–tubulin dimers are termed protofilaments, 13 of which associate laterally to make up the microtubule (Fig. 1B). This polarized arrangement of tubulin dimers extends into a supra-molecular polarity with an α-tubulin exposed ‘minus-end’ and a β-tubulin exposed ‘plus-end’.

Microtubules are constantly switching between phases of polymerization and depolymerization, a process known as dynamic instability.^101^ This feature forms the basis for the ability of cells to swiftly remodel their microtubule network in response to intracellular or extracellular cues. In most cells, this dynamic behavior is only observed at microtubule plus-ends, since the minus-ends are most often stably embedded into the microtubule-organizing center (MTOC) from which microtubules nucleation is promoted. Centrosomes function as the major MTOC during mitosis, whereas during interphase significant microtubule-nucleation can be observed from other structures including the Golgi apparatus.^125^ The molecular mechanisms underlying microtubule nucleation have recently been described in an excellent review and will therefore not be covered here.^77^

### Biochemistry of tubulin

The tubulin protein family contains 3 main members in eukaryotes: α-, β-, and γ-tubulin, each being approximately 55 kDa in size.^112^ The majority of γ-tubulin is organized in γ-tubulin ring complexes (γ-TuRC) in the MTOC, where it stabilizes the microtubule minus-ends and acts as a microtubule nucleation template.^77^ The tubulin heterodimers that make up the microtubule lattice are composed of one α- and one β-tubulin subunit. The most common form of microtubules *in vivo* consists of 13 protofilaments and is ordered into a so-called B-lattice, which is characterized by homotypic lateral contacts resulting in α-α and β-β interactions (Fig. 1C). Due to an axial offset between the protofilaments, the lattice contains a helical twist that gives rise to a discontinuity, known as the seam. This seam runs along the length of the microtubule and contains lateral (heterotypic) α-β interactions^110^ (Fig. 1C).

The α-tubulin subunit contains a non-exchangeable GTP nucleotide that is buried at the α-β interface (at the so-called ‘N-site’) (Fig. 1A). In contrast, the β-tubulin subunit accommodates an exchangeable GTP nucleotide (at the ‘E-site’). The presence of GTP at this site allows a new dimer to bind longitudinally with a heterotypic interaction at the exposed plus-end.^111^ After the incorporation of a tubulin dimer into the microtubule lattice, the GTP nucleotide at the E-site becomes hydrolysed, resulting in a lattice containing mostly GDP nucleotides at the E-site^34^ (Fig. 1D).

In cryo-electron microscopy (cryo-EM) studies, the ends of growing and shrinking microtubules adopt different structural configurations. Growing microtubule plus-ends contain mostly straight protofilaments, while shrinking plus-ends peel outward after having lost the lateral interactions between its protofilaments^94^ (Fig. 1D, E). This observation introduced the early hypothesis that GTP-tubulin is relatively straight whereas GDP-tubulin is more curved. However, recent work has shown that both GTP- and GDP-bound dimers have similar curvatures in solution.^9^ It is only after incorporation of GTP-tubulin into the microtubule lattice that a more straight conformation is adopted.^106^ A recent comparison of GDP- and GMPCPP- (a slowly hydrolysable GTP-analog) bound microtubules showed that GTP hydrolysis promotes a major change in the tubulin dimer, characterized by a compaction of the E-site and strain introduction into the microtubule lattice.^6^

### The GTP cap

It is hypothesized that the time-delay between incorporation of a tubulin dimer into the microtubule lattice and its subsequent GTP hydrolysis generates a ‘GTP cap’ at the growing plus-end of a microtubule (Fig. 1D). GTP hydrolysis is fundamentally coupled to microtubule (in)stability,^23^ but is not essential for microtubule polymerisation, as microtubule growth occurs normally in the presence of GMPCPP.^66^ The GTP cap is capable of preventing the GDP lattice from releasing the strain build-up in the lattice.^23^ When the GTP cap is lost, the labile GDP-rich lattice follows the conformational trajectory toward a curved structure (Fig. 1E). This results in the loss of lateral contacts between the individual protofilaments and subsequent microtubule depolymerisation. The precise size and state of this protective GTP cap is however still a matter of debate. Early studies showed that both the plus- and minus-ends of microtubules are stabilised by a short region of about 200 GTP-bound tubulin dimers.^147^ Although subsequent experiments with GMPCPP-stabilised microtubules suggested that a single monolayer of tubulin dimer is sufficient to stabilize microtubules,^22^ recent evidence suggests that the GTP cap does not exist as a monolayer^126^ and can even extend to about 750 tubulin subunits *in vivo*.^130^ Fluctuations in the rate of α/β-tubulin incorporation at the microtubule plus-end, together with the stochastic nature of GTP hydrolysis, results in a dynamic GTP cap.^64^ As a consequence, faster growing microtubules accumulate a larger GTP cap compared to slowly growing microtubules, and are therefore less prone to undergo catastrophe.^39^

### Dynamic instability

Dynamic instability, the alternation between phases of growth and shrinkage, can be characterized by a number of parameters: 1) the rate of growth, 2) the rate of shrinkage, 3) the catastrophe frequency (the rate of switching from a growing to a shrinking state), and 4) the rescue frequency (the rate of switching from a shrinking to a growing state).

The microtubule growth rate is linearly dependent on the soluble tubulin concentration, whereas the shrinkage rate is constant.^146^ Since microtubules do not elongate at a constant rate, it has been proposed that variability in the growth rate results in fluctuations in the length of the dynamic GTP cap.^64,74,126^

Microtubule catastrophe was originally assumed to be a single-step stochastic event that is mediated by GTP hydrolysis.^32^ However, it was later established that microtubule catastrophe is likely a multi-step process^15,49,97,113^ and that microtubule “aging” is responsible for the nonexponential distribution of microtubule lifetimes observed *in vitro*.^158^ In cells, the combination of timely and spatially regulated MAPs^50,143^ and the physical contact with cellular structures^68,135^ control the microtubule catastrophe frequency (see “Modulating microtubule dynamics”).

In contrast to the aforementioned parameters, the biochemical and biophysical principles underlying the switch from microtubule shrinkage to growth (rescue) are still poorly understood. Early studies have shown that microtubule rescue frequency shows no strong correlation with tubulin concentration.^146^ It has been suggested that patches of unhydrolyzed GTP in the GDP lattice (Fig. 1E) can disrupt microtubule depolymerisation and initiate a rescue event.^34^ Besides these GTP islands, certain MAPs such as CLASP can promote rescue events through interaction with the microtubule lattice^4^ although this is mechanistically poorly understood.

### Single microtubule approaches for studying microtubule dynamics

Studying dynamic instability with microtubules that have a sub-diffraction-limited width of 25 nm can be a challenge both *in vivo* and *in vitro*. Pioneering experiments using dark field microscopy to observe single microtubules^101^ were followed up by video-enhanced differential interference microscopy (VE-DIC),^146^ and epi-fluorescence techniques.^143^ With the advent of fluorescence speckle microscopy (FSM),^150^ it also became possible to observe the dense network of microtubules in cells. Later, the use of total internal reflection fluorescence (TIRF) microscopy techniques allowed for high temporal resolution imaging with high signal-to-noise ratios due to the elimination of fluorescence background arising from the free tubulin in solution.^12,17^ TIRF microscopy also enables experiments using multiple fluorophores, which allows simultaneous measuring of both microtubule dynamics and MAP localization.^51^ Finally, fluorescence recovery after photo-bleaching (FRAP) techniques now enable the investigation of turnover times of MAPs on the microtubule lattice plus-end.^38^

## Modulating microtubule dynamics

The variety of different biological processes that the cytoskeleton needs to adapt to in cells requires continuous and extensive remodeling of the microtubule network. The MTOC acts as a nucleation template for microtubules and stabilizes minus-ends,^77^ allowing for about 65% of the total tubulin content in cells to be polymerized.^160^ The dynamic plus-ends explore the cytoplasm by constantly switching between phases of growth and shrinkage, allowing for interactions with other cellular structures at different locations in the cell. When cells enter mitosis however, the microtubule network undergoes a major transformation into a highly characteristic structure called the ‘mitotic spindle’, which is required for chromosome segregation. Extensive remodeling of the microtubule network is mostly mediated by MAPs, which can spatially and temporally alter the dynamic properties of microtubules.^14^

### Tubulin isotypes and modifications

In the past decades, multiple ways for a cell to control microtubule dynamics have been uncovered. Firstly, regulation of β-tubulin isotype expression levels generates heterogeneous microtubules^67^ that could be capable of exhibiting different characteristics. However, until now, only a few highly specialized microtubule types such as neuronal microtubules^70^ and cilliary axonemes^122^ have been shown to be composed of specific β-tubulin isotypes.

Secondly, several post-translational modifications (PTMs) have been shown to modify the stability and structure of the microtubule lattice.^58,67^ Most PTMs affect polymerised microtubules and are not acquired on soluble tubulin.^144^ For this reason, PTMs have only been observed *in vivo* on long-lived subpopulations of microtubules. These modifications may either act individually or in concert with each other by creating a combinatorial readout.^121,141^ It has been proposed that different tubulin isotypes or PTMs, known as the ‘tubulin code’, can result in unique interactions with MAPs.^144^ Detyrosination, for example, which refers to the enzymatic removal of the C-terminal tyrosine of α-tubulin subunits,^57^ can have extremely different outcomes in various biological systems. 1) It has been shown that the localization and activity of several proteins that affect dynamic instability are directly affected by microtubule detyrosination.^118,119^ 2) Recent studies revealed that detyrosination decreases the processivity of the motor protein kinesin-1, but causes the opposite effect on kinesin-2 and −7 motors.^10,131^ 3) Microtubule detyrosination promotes their interaction with the sarcomere (via desmin) to regulate cardiac myocyte stiffness and contractility.^123^ Whether these biological outcomes are a result of the cell specific regulation of (the expression of) downstream effectors of this PTM or whether detyrosination triggers different outcomes in combination with additional PTMs is currently unknown.

### Microtubule-associated proteins (MAPs)

MAPs play a central role in adapting microtubule dynamics in order to accommodate the large variety of functions microtubules have to perform within a cell. MAPs are capable of altering various parameters of microtubule dynamics, either through direct microtubule contact or through the association with other MAPs. CLIP-170 was the first MAP observed to accumulate at growing microtubule plus-ends *in vivo*. It was observed that CLIP-170 binds in stretches along microtubule ends, forming comet-like structures.^117^ An important subclass of MAPs comprises so-called plus-end tracking proteins (+TIPs), which are capable of accumulating specifically at the growing plus-ends of microtubules.^129^ Their localization to microtubule plus-ends makes them prime candidates for establishing interactions with other intracellular structures while growing microtubules explore the cytoplasm. Since this first discovery, many +TIPs have been identified and their contribution to a large +TIP interaction network has been established (see “The end binding protein family” and “The + TIP interaction network”) (for excellent recent reviews, see refs. 1, 47.

### The end binding protein family

A central player in the +TIP interaction network is the conserved family of End Binding (EB) proteins. The first *in vitro* reconstitution of microtubule plus-end tracking showed that Mal3, the EB protein from fission yeast, is able to autonomously track growing microtubule ends.^12^ Three subtypes of mammalian EB proteins have been described: EB1, EB2, and EB3. EB1 and EB3 have been found to increase microtubule polymerisation rate both *in vivo* and *in vitro*.^97^ In addition, EB proteins increase the microtubule catastrophe rate *in vitro*.^79^ by destabilizing the GTP cap.^39,162^ Interestingly, *in vivo* depletion of EB1 and EB3 results in a reduction of persistent microtubule growth and in an increase of catastrophe frequency, resulting in fewer microtubules being located near the cell cortex.^79^ The discrepancy between these *in vitro* and *in vivo* observations is most likely the consequence of the removal of other EB dependent +TIPs that impact on microtubule dynamics.^47^

The interaction of EB with microtubule plus-ends is mediated by its N-terminal calponin homology (CH) domain^132^ and does not require dimerization.^19,79^ It has been demonstrated that EB can bind to the lattice of microtubules stabilised by GTPγS (a slowly hydrolysable GTP analog) with similar high affinity as to the microtubule tip, but not to GMPCPP-stabilised microtubules.^96^ Taking into account the intermediate curvature of GMPCPP microtubules,^105^ (compared to the straight lattice and the curved tubulin dimers, the view emerged that GMPCPP mimics the unhydrolysed GTP-state located at the very end of the microtubule tip, while GTPγS mimics an intermediate (GDP-Pi) nucleotide hydrolysis state.^16^ This model is further substantiated by the fact that the position of the EB comet is displaced up to ∼100nm from the tip.^39,97^

Recent high-resolution cryo-EM studies have now elucidated the specific interactions between EB3 proteins and the microtubule lattice and propose an explanation for their effect on microtubule dynamics. The CH domain of EB binds at the interface of 4 tubulin dimers, except at the seam, and is therefore positioned in close proximity to the nucleotide at the E-site.^98^ Once bound, EB promotes conformational changes in α-tubulin leading to a compacted lattice with a unique twist. As a consequence, the catalytic residue in the α-subunit of the longitudinally adjacent dimer is brought into closer proximity to the E-site, facilitating GTP hydrolysis and promoting microtubule catastrophe.^6^ Since EB binds at the intersection of lateral and longitudinal contacts, it also promotes seam closure and stabilises the end structure, resulting in an increased microtubule growth rate.^162^

### The +TIP interaction network

The +TIP interaction network is build around the EB proteins, which can autonomously recognize the plus-end of a growing microtubule.^97,162^ Upon plus-end binding, EB proteins create a platform for recruiting a multitude of other MAPs through several interaction modules.^1^

A coiled-coil region at the C-terminus of EB is required for its dimerization and mediates the interaction with other +TIPs through the EB homology (EBH) domain. Interactions with the EBH domains are mostly mediated by a microtubule tip localization signal (MtLS), which is characterized by a short Ser-x-Ile-Pro (SxIP) motif that is embedded in a region containing basic residues.^61^ Examples of +TIPs containing this SxIP motif are the microtubule-stabilizing family of CLASP proteins,^37^ the microtubule-actin crosslinking factor (MACF),^89^ and the microtubule-destabilizing kinesin MCAK.^33^ In addition to the EBH domain, the flexible C-terminal tail of EB contains an EEY/F sequence capable of binding to CAP-Gly domains present in the microtubule-rescue promoting CLIP-170^80^ and p150^glued^, which is part of the dynactin complex.^128^ The EEY/F sequence is also present on CLIP-170 itself, where it mediates an auto-inhibitory interaction with its own CAP-Gly domain,^46^ and on α-tubulin, suggesting copolymerization of tubulin and CLIP-170 onto the microtubule end.^100^

Besides EB, other +TIPs are also capable of interacting with the microtubule lattice and tip directly. The two structurally related proteins XMAP215 (homolog of human ch-TOG) and CLASP are recruited to the plus-end by conserved TOG domains. It has been shown that XMAP215 binds tubulin in a 1:1 complex and catalyzes the addition of up to 25 dimers to the growing plus-end.^17^ *In vitro*, XMAP215 acts synergistically with EB to increase the growth rate to physiological levels.^159^ CLASP on the other hand promotes microtubule rescue and supresses catastrophe events.^3^ Additionally, motor proteins are also capable of tracking the microtubule ends. The depolymerizing kinesins Kip3 and MCAK have different effects on microtubule dynamics: Kip3 slows down microtubule growth in a length-dependent manner and MCAK eliminates microtubule aging altogether, transforming microtubule catastrophe a single-step process.^49^ Another example of a motor protein capable of altering microtubule stability is cortical dynein, which can capture incoming microtubule plus-ends and trigger a catastrophe.^85^

## Pushing forces generated by microtubule polymerization

**Figure 2.**
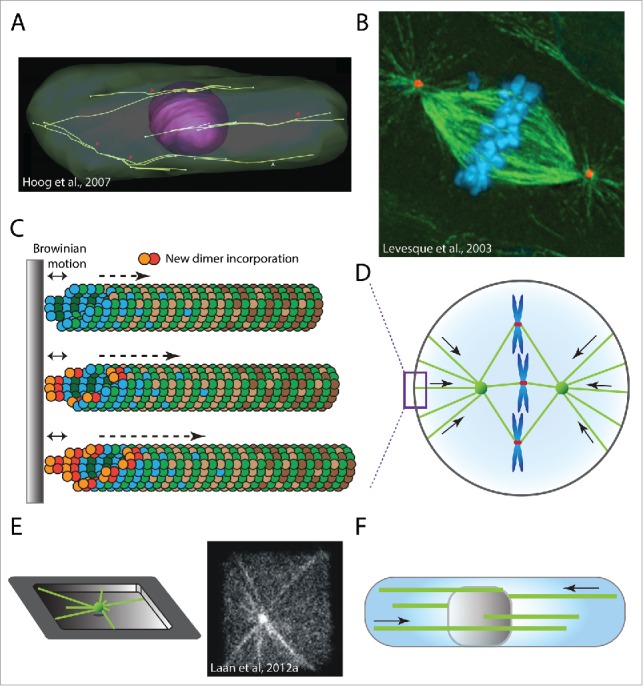
Pushing forces generated by polymerising microtubules. (A) 3D model of interphase microtubule organization in fission yeast, image from ref. 62. (B) Mitotic spindle in human CFPAC-1 cell, image from ref. 90. showing DNA in blue, centrosomes in red and microtubules in green. (C) Microtubule growing against a rigid object slows down microtubule growth. Brownian motion generates space between the microtubule plus-end and the object and allows for the slow incorporation of new tubulin dimers (red/orange). (D) Schematic representation of a eukaryotic mitotic cell, showing centrosomes (green spheres), microtubules (green lines), chromosomes (blue) and kinetochores (red) and the microtubule pushing forces (arrows) that act on the mitotic spindle. (E) Reconstitution of microtubule aster positioning in microfabricated chambers, displaying a schematic representation (left) and immunofluorescent image showing fluorescent tubulin (right).^85^ (F) Nuclear positioning in fission yeast by microtubule pushing forces.

Dynamic microtubules are able to exert forces on interacting entities. Growing microtubules can generate forces by simply colliding with membranes, organelles or protein(−complexe)s, but are also capable of promoting directed transport through the interaction of plus-end tracking complexes with their cargo partners. The importance of microtubule pushing forces has long been appreciated in various biological systems, most notably in mitotic spindle formation and positioning (Fig. 2B) (see “Positioning the nucleus and mitotic spindle”). A wide variety of studies now starts to unveil a more diverse role for microtubule pushing forces, visible through their importance in cell polarity, directed protein transport, organelle architecture and the arrangement of other cytoskeletal networks (see “Microtubule pushing forces in organelle architecture”).

### Positioning of the nucleus and mitotic spindle

In some organisms, like yeasts, the MTOC (in this case the spindle pole body) is an integral part of the nuclear envelope throughout most of the cell cycle. This results in the concerted positioning of the nucleus and the mitotic spindle. In the fission yeast *S. pombe*, the position of the interphase nucleus is an important determinant for the position of the cleavage plane during mitosis.^25,136,138^ (Fig. 2A, F). Within these bundles, microtubules are organized in an antiparallel fashion with their growing plus-ends oriented toward the cell poles.^62,69,139^ These growing microtubule plus-ends collide with the plasma membrane at the cell tips^62^ where they exert pushing forces that result in nuclear displacement^136,137,139^ (Fig. 2A, F). A balance between the pushing forces generated at opposite sides of the cell has been suggested to ensure a centralised localization of the nucleus,^114,137^ even during cell elongation in interphase.^30^ This nuclear centring is not only achieved by balancing the number of microtubules that push on either side, but also by active regulation of microtubule dynamics by PRC1 (ase1 in *S. pombe*),^30,69,92^ CLIP170 (tip1 in *S. pombe*),^18,30^ and EB3 (mal3 in *S. pombe*).^11^

Recent evidence from *Drosophila* oocytes suggests that similar microtubule pushing forces are responsible for anterior-directed motion of the oocyte nucleus in order to establish the dorso-ventral axis.^163^ In contrast to the microtubule bundles observed in fission yeast,^139^ nuclear positioning in *Drosophila* oocytes is mediated by the pushing forces generated by approximately 6 singular microtubules at any given time.^163^

Centrosome positioning depends on force-generating microtubules in virtually all studied organisms, but whether these microtubules are pushing or pulling is organism specific. Up to now, only limited evidence supports a clear contribution of microtubule pushing forces in MTOC positioning in non-yeast cells. Since most eukaryotic cells are significantly larger than yeast cells, they are expected to have difficulties to efficiently transmit pushing forces as a result of microtubule buckling.^35,36,63^ It is therefore thought that larger cells mostly rely on microtubule pulling forces for efficient centrosome positioning^103^ (see “Centrosome positioning by cortical anchors” and “Dynein-mediated centrosome positioning”).

### Microtubule pushing forces in organelle architecture

Microtubules play an important role in intra-cellular organization and in defining organelle shape. In addition to the formation of lateral contacts between the microtubule lattice and membrane-enclosed compartments, microtubule plus-ends and membranes can also form dynamic connections that can reshape or position a wide variety of intracellular structures.^56^ In *Xenopus* egg extracts, growing microtubule plus-ends can interact with membranes and push out long, extended endoplasmic reticulum (ER-)tubes.^149,151^ The tips of ER-tubes have also been observed to track growing microtubule plus-ends in human tissue culture cells, a process that is mediated by an interaction between EB1 and the ER transmembrane protein STIM1.^52,152^

In *S. pombe*, mitochondrial tubules that interact with microtubule plus-ends can shrink and extend in a coordinated motion together with microtubule (de)polymerization.^155^ This process does not dependent on microtubule motor-proteins^91^ and is thought to promote the motility and distribution of mitochondria.^91,155^ At present, the influence of microtubule pushing forces on organelle shape and positioning is relatively understudied and it is therefore unknown to what extent these processes are conserved and important for cellular fitness.

## Reconstituting microtubule pushing forces

The biological relevance of the pushing forces generated by growing microtubules has been appreciated for several decades. This has stimulated the extensive biophysical characterization and theoretical modeling of the force-generating capacity of growing microtubule plus-ends (see “Biophysical principles behind microtubule pushing forces”). In addition, significant efforts have been made to reconstitute the cellular processes that depend on microtubule pushing forces using *in vitro* bottom-up approaches. These efforts are largely focused on the reconstitution of MTOC positioning by microtubule pushing forces and will be discussed in see “Biophysical principles behind microtubule pushing forces.”

### Biophysical principles behind microtubule pushing forces

Growing microtubules are able to generate substantial pushing forces, resulting in the displacement or deformation of movable or flexible obstacles (assuming the MTOC position is fixed). On the other hand, when the obstacles are immobile and rigid, the growing microtubule can push itself away from the obstacle (assuming the MTOC position is not fixed). Microtubule pushing forces are generated by the continued incorporation of tubulin-dimers at the plus-end of a growing microtubule. According to the ‘Brownian ratchet’ model, thermal fluctuations in the position of the growing microtubule and the obstacle create large enough gaps to accommodate the addition of new tubulin dimers^142^ (Fig. 2C). This enables the continued elongation of the microtubule plus-end (albeit at a lower velocity) and the generation of a pushing force. To investigate the forces generated by growing microtubules, optical tweezers have been employed to trap a microtubule-bound dielectric bead with a focused laser.^73^ Interaction of the growing microtubule with a barrier results in a measureable displacement of the bead, enabling the determination of the generated pushing force.^74^ The maximum pushing force (*Fc*) that an individual microtubule can generate through continued tubulin-subunit incorporation is in the range of 3-4 pN.^36,68,78^ This maximum pushing force is a function of both microtubule-length (*L*) and rigidity (κ), and can be expressed as *Fc = 2*πκ*/L*^*2*^.^36,75,83^ Upon encountering a rigid barrier, growing microtubules can buckle when the exerted force is greater than the critical buckling force (the force beyond which a microtubule buckles).^28,36,63^ Since the critical buckling force decreases with the square of microtubule length, long microtubules are able to generate significantly lower pushing forces compared to short microtubules.^36^ However, as the cellular context wherein microtubules grow can affect their rigidity, growing microtubules might be able to generate significantly different amounts of force *in vivo* than their length-force relationship would predict. In addition, microtubules often associate into microtubule bundles, which increases their force-generating capacity in an additive fashion.^84^

### Reconstituting centrosome positioning

Since the amount of force that can be generated by a growing microtubule depends on its length, the shape and position of the mitotic spindle is strongly influenced by the geometrical confinement in which these spindles assemble. This idea is supported by mechanical models that describe how microtubule pushing forces mediate aster positioning in both 2D- and 3D-confinements.^60,63,85,93,116^ Purified centrosomes are able to nucleate microtubules in the presence of tubulin and GTP.^102^ The forces generated by these growing microtubules are sufficient to promote self-organization of microtubule-asters in the center of squared or round micro-fabricated chambers^60,85,107^ (Fig. 2E). Once microtubules grow longer, this geometric symmetry is broken, resulting in a decentred microtubule aster position.^60,85^ The decentralised position of these microtubule asters is relatively stable, although a central position can be regained by increasing microtubule catastrophe rates.^41^ Recent developments using microfluidics now allow the first reconstitutions of mitotic spindle assembly and positioning in spherical emulsion droplets that mimic the geometrical confinement of a mammalian mitotic cell^124,145^ (see also “Geometrical confinements”).

## Pulling forces generated by microtubule depolymerization

In order for a depolymerizing microtubule to transmit a pulling force to its potential cargo, the cargo must be capable of forming and maintaining load-bearing attachments to a rapidly shrinking microtubule. The most extensively studied examples of microtubule polymerization-driven force-generation come from mitosis. Mitotic spindle formation and positioning are not only dependent on microtubule pushing forces, but also rely on stable links between the cell cortex and depolymerizing microtubules (see “Microtubule attachment sites at kinetochores”). In addition, mitotic chromosomes have to form stable interactions with spindle-microtubules in order to become equally distributed over the 2 new daughter cells. The physical separation of the replicated sister-chromatids is driven by the formation of connections between a specialized chromatin-bound protein complex, called the ‘kinetochore’, and depolymerizing microtubules originating from opposing centrosomes (see “Centrosome positioning by cortical anchors”). Both cortical- and kinetochore-localized protein-complexes that interact with microtubule plus-ends are able to directly modulate the dynamic instability properties of these microtubules (see “Modulating microtubule dynamics in mitosis”).

### Centrosome positioning by cortical anchors

**Figure 3.**
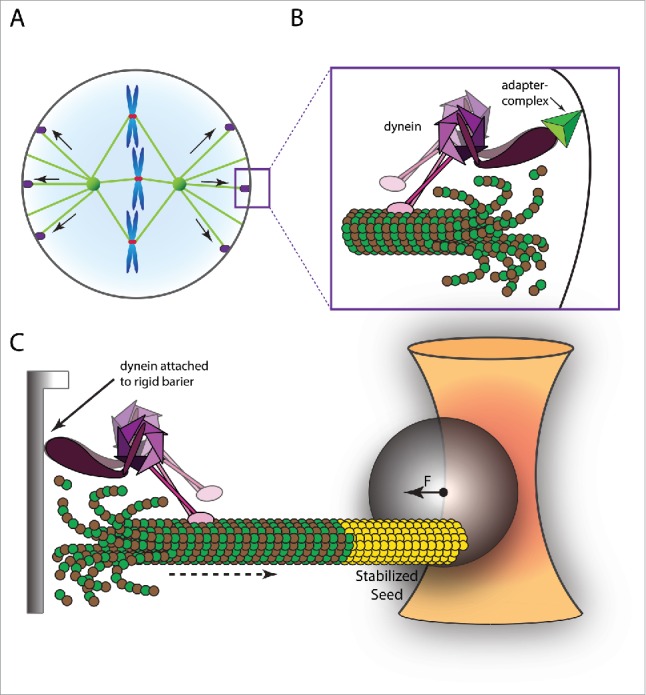
Pulling forces generated by depolymerising microtubules drive spindle positioning. (A) Mitotic spindle positioning is accomplished by linking depolymerizing microtubules to the cell cortex. (B) Dynein (purple) is anchored to the cell cortex by means of the NuMa/LGN/Gαi trinary adapter complex (green) and interacts with shrinking microtubules. (C) Single molecule approach to study dynein-mediated pulling forces using an optical trap setup. Barrier (gray) attached dynein is bound to a depolymerizing microtubule that has grown from a stabilised seed (yellow) that has been linked to a magnetic bead (gray sphere). The formation of load-bearing attachments results in a pulling force (F) that displaces the bead from the optical trap (yellow).

Whereas microtubule pushing forces are the main forces underlying spindle pole body and nucleus positioning in yeast, larger eukaryotic cells usually depend on microtubule pulling forces for centrosome positioning and spindle architecture (Fig. 3A). Cortical microtubule anchors are capable of converting the force generated by microtubule depolymerization into a pulling force on the centrosome. Cytoplasmic dynein (hereafter referred to as ‘dynein’), a minus-end directed microtubule motor, appears to play a central role in centrosome positioning both during interphase and mitosis.

During interphase, dynein localized at the cell cortex is responsible for centrosome positioning^20^ and promotes the reorientation of the MTOC toward the immunological synapse in antigen-stimulated T cells.^95,157^ During cell migration, enrichment of dynein at the leading edge of a migrating cell is required for cell polarity, MTOC positioning and cell motility.^40,48,115^

In mitosis, cortical dynein functions as a force generating and/or force transmitting linker between microtubules and the plasma membrane in many organisms^24,85,108^ (Fig. 3B). In budding yeast, cortical dynein forms lateral attachments to spindle microtubules and relies on its minus-end directed motor activity to slide the microtubules along the long axis of the cell.^104^ In metazoans, dynein is recruited to the cell cortex by the heterotrimeric Gαi/LGN/NuMA complex.^82^ In this case, cortical dynein pulls on centrosomes by forming load-bearing interactions with depolymerizing microtubule plus-ends^24,85,108^ (Fig. 3C). In specialized cases, asymmetry in dynein-mediated force generation at the cell cortex is also thought to control asymmetric spindle position, like in the *C. elegans* one cell stage embryo.^53,108^

In addition, early studies using a chemical inhibitor of dynein's ATPase activity have shown defects in pole-ward movement of centrosomes during late anaphase (a process known as spindle elongation) in vertebrate cells.^13,21^ This movement was later shown to depend on microtubule pulling forces generated by cortically anchored dynein in a wide variety of organisms.^42,76,108,164^

### Microtubule attachment sites at kinetochores

**Figure 4.**
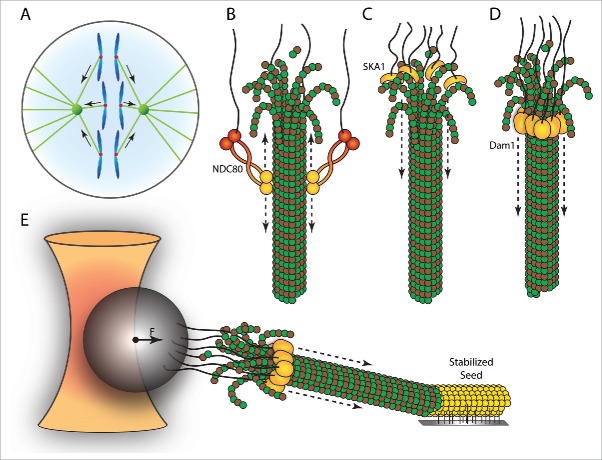
Pulling forces generated by depolymerising microtubules drive chromosome segregation. (A) Mitotic cell in anaphase showing the released sister chromatids that are pulled toward the opposing centrosomes. (B-D) Different modes of kinetochore-microtubule attachments, showing lateral attachment of the conserved NDC80-complex to the microtubule lattice (B), ring-formation of the Dam1-complex around a microtubule (C) and attachment of the SKA1-complex to curved protofilaments (D). (E) Single molecule approach to study kinetochore-mediated pulling forces using an optical trap setup. A microtubule growing from an immobilised microtubule seed (yellow) attaches to kinetochore-complexes or –particles (here indicated the Dam1 complex). Microtubule depolymerisation in the presence of load-bearing attachments results in a pulling force that displaces the bead (gray sphere) from the optical trap (yellow).

Microtubules emanating from opposing centrosomes attach to sister chromatids in a bipolar fashion in order to promote faithful chromosome segregation (Fig. 2B). Mitotic kinetochores are composed of over 80 different proteins and are essential for forming load-bearing attachments between the chromatin and spindle microtubules.^43^ After stable attachments have been established between kinetochores and spindle microtubules, the sister chromatids physically separate by tracking depolymerizing microtubules^81^ (Fig. 4A). The most broadly conserved microtubule-attachment site at the kinetochore is the 9-subunit KNL1/MIS12-complex/NDC80-complex (KMN) Network. Both in yeasts and in vertebrates, the KMN Network is accompanied by an additional microtubule-binding complex: the Dam1- or SKA1-complex respectively. Although all 3 complexes can independently form load-bearing attachments to microtubules, the mode with which they interact is fundamentally different.

The KMN Network contains microtubule-binding regions in both the NDC80-complex and KNL1,^27^ the former of which is the most extensively studied component. The NDC80-complex binds to microtubules at the α/β-tubulin interface and can diffuse along the microtubule lattice.^7,120^ Although the NDC80-complex can form load-bearing attachments to microtubules, it has only a weak ability to track depolymerizing microtubule plus-ends^120,127^ (Fig. 4B). Both the Dam1- and SKA1-complex are recruited to kinetochores by the NDC80-complex and provide the NDC80-complex with the ability to track depolymerizing microtubule ends.^87,127,134^ The SKA1-complex also binds to the microtubule lattice but is unique in its ability to stably interact with both straight and curved microtubule protofilaments^127^ (Fig. 4C). The 10-subunit Dam1-complex adopts a ring-like structure around the microtubule that is thought to slide along the microtubule lattice using the force generated by the outward pealing of protofilaments during microtubule depolymerization^54,148,154^ (Fig. 4D).

### Modulating microtubule dynamics in mitosis

During mitosis, many events require the controlled growth and shrinkage of microtubules. Stable kinetochore-bound microtubule bundles are composed of both growing and shrinking microtubules.^8^ In metaphase, pole-to-pole oscillation of sister-chromatids correlates with coordinated bursts of microtubule growth.^8^ Inactivation of the cyclin-dependent kinase 1 (CDK1)-cyclin A complex stabilises these kinetochore-fibers when all chromosomes are aligned at the cell equator.^71^ Next, during anaphase, kinetochore-bound microtubule bundles collectively undergo catastrophe in order to drive sister chromatids toward opposite centrosomes. This process is still poorly understood at the molecular level, but might be explained as a consequence of the loss of tension between sister-kinetochores that stabilised these microtubules during metaphase.^2^ During late anaphase (also called ‘anaphase b’), the mitotic spindle elongates through the pole-ward movement of the opposing centrosomes in order to accommodate complete segregation of the replicated chromosomes. During this process, the mitotic spindle drastically reorganises with the majority of growing plus-ends localizing to the inter-polar region, in both *Drosophila* and yeast cells.^26,31,45^ It has been suggested that a spatial gradient of microtubule catastrophes could account for this spindle reorganization and elongation,^26^ although at present there is no molecular understanding of this phenomenon.

## Reconstituting microtubule pulling forces

Over the past decade, significant advancements have been made in the purification and biochemical characterization of microtubule-binding protein complexes. Since most of these proteins are unstable when separated from their biochemical context, determining their stoichiometric composition has been essential for reconstitution-based assays. This, together with the rapid development of methods to measure force-resistance of protein-protein interactions using optical tweezers setups, has provided important insights at the single-molecule level. In addition, more and more efforts are being made to reconstitute the functions of these protein complexes in cell-like geometrical confinements.

### Biophysical principles behind microtubule pulling forces

In addition to the ability of microtubules to generate pushing forces by growing into obstacles, the energy released by microtubule depolymerization can be used to generate pulling forces. The forces associated with microtubule depolymerization are in the range of 30-65 pN^32,55^ and are therefore about an order of magnitude larger than the forces generated by microtubule growth (3-4 pN).^36^ Microtubule catastrophe can be actively promoted or inhibited by MAPs and physical barriers (see “Introduction to microtubule dynamics”). Over long distances, microtubule-mediated pushing forces might be less efficient than pulling forces due to length-dependent microtubule buckling (see “Biophysical principles behind microtubule pushing forces”). In the case of mitotic spindle positioning, smaller cells (like yeasts) usually rely on microtubule pushing forces, whereas the pulling forces generated by depolymerizing microtubules drive the same process in metazoa.

### Single-microtubule force measurements on kinetochore structures

The ability to track depolymerizing microtubules has been investigated *in vitro* for a wide range of proteins. The complexity of the molecules tested in these setups varies from single proteins (dynein heavy-chain)^85^ and (combinations of) recombinant protein complexes (NDC80-, Dam1-, SKA1-complexes)^120,127,153,154^ to kinetochore particles^2^ (Fig. 4E). The maximum load-bearing force measured for individual microtubule binding complexes is about 2-3 pN (Table 1), whereas purified kinetochores from budding yeast can resist a load-bearing force up to ∼11 pN.^2^ These numbers agree well with pioneering studies in cells, which have estimated that kinetochores can carry loads of 1-10 pN per microtubule.^5,109^ It is important to note that the different microtubule binding complexes are not conserved in all eukaryotes and they can be present at kinetochores in different stoichiometries. Whereas a single Dam1-complex can entrap a single microtubule,^148^ the SKA1-complex forms dimers,^153^ and the NDC80-complex undergoes extensive oligomerization, which is suggested to lead to additive or even cooperative microtubule binding.^7^ It is however unknown to what extent all complexes form independent load-bearing attachments at the same time, making it difficult to assign individual activities to single complexes *in vivo*.

**Table 1.** Maximum rupture force measurements of microtubule sub-complexes.

	Maximum rupture force	References
NDC80-complex	2.5 pN	^120^
	2.7 pN	^134^
Dam1-complex	3.0 pN	^165^
	2.3 pN	^54^
	3.2 pN	^166^
SKA1-complex	N.D.	
NDC80 + Dam1	4.4 pN	^134^
NDC80 + SKA1	N.D.	
Kinetochores	11 pN	^2^

*Note*. N.D., non-determined

### Dynein-mediated centrosome positioning

In optical tweezers setups, dynein can form load-bearing attachments to the plus-ends of depolymerizing microtubules and resist forces up to 5 pN^59,72,85,86^ (Fig. 3C). Unlike the extensive studies on kinetochore-microtubule attachments (see “Single-microtubule force measurements on kinetochore structures”), the molecular mechanism by which dynein attaches to depolymerising microtubules remains largely elusive. For instance, it is at present unknown whether stable dynein-mediated attachments are formed by individual molecules or depend on a number of cooperating motor proteins. Do several dynein-molecules form a ring-like structure (such as is the case with the Dam1-complex)? Can dynein interact with curved protofilaments (such as is the case for the SKA1-complex)? Or are different mechanisms involved? And how does this relate to dynein's minus-end directed motor activity?

Several *in vitro* studies are now building toward increasingly complex *in vivo*-like reconstitutions of the function of cortical dynein in mitotic spindle formation and positioning^85,124,145^ (see “Reconstituting complex force-generating microtubule systems”). In micro-fabricated 2D-chambers, the pushing forces generated by growing microtubules are sufficient to promote centrosome displacement to the periphery of the chamber (see “Reconstituting centrosome positioning”). However, when the walls of these chambers are coated with dynein, centrosomes are stabilised in a more central position, even after extensive microtubule growth.^85^ This effect can be reconstituted in spherical (3D) water-in-oil emulsion droplets, where lipid-anchored dynein also generates a centring force on astral microtubules.^124,145^

### Modulating microtubule dynamics through end-on interactions

In addition to the ability to hold on to depolymerizing microtubules, many microtubule-binding complexes at the kinetochore also actively control microtubule dynamics. Purified kinetochores from *S. cerevisiae* cannot only form load-bearing attachments to shrinking microtubules, but they also reduce the microtubule catastrophe rate when these attachments are under tension.^2^ Likewise, recombinant Dam1- and NDC80-complexes reduce catastrophe rates and promote rescue events *in vitro*.^44,140^ In contrast, the SKA1-complex induces the formation of and interacts with curved protofilaments.^127^ Although this is expected to promote microtubule catastrophes (see “Introduction to microtubule dynamics” and “Modulating microtubule dynamics”), there is currently no direct evidence supporting this idea.

Both in budding and fission yeast, dynein-mutant cells have significantly longer microtubules^24,156^ and barrier-coated dynein has been suggested to increase microtubule catastrophe rates *in vitro*.^85^ Interestingly, binding of dynein to depolymerising microtubule plus-ends slows down microtubule shrinkage by straightening and thereby stabilizing protofilaments in an ATP-dependent fashion.^59,85^ Similar to dynein, a cortex-localized pool of *Drosophila* Dm-Kat60 (a functional ortholog of katanin) also interacts with microtubule plus-ends and promotes microtubule catastrophes.^161^ Although cortical Dm-Kat60 and dynein seem to have a similar function in regulating cell polarization and migration,^161^ it is unknown whether Dm-Kat60 also promotes this by forming load-bearing interactions with depolymerizing microtubules.

### Reconstituting complex force-generating microtubule systems

The studies described above have proven to be extremely valuable for our molecular and biophysical understanding of force generation by growing and shrinking microtubules. However, most of these studies have been executed in relatively simplified systems, either using force-measurements on individual microtubules or by studying the positioning of a single centrosome in simple geometrically confinements. Here we discuss recent progress on reconstituting more complex and *in vivo*-like microtubule force-generating systems, with a focus on mitotic spindle assembly and positioning.

## Geometrical confinements

**Figure 5.**
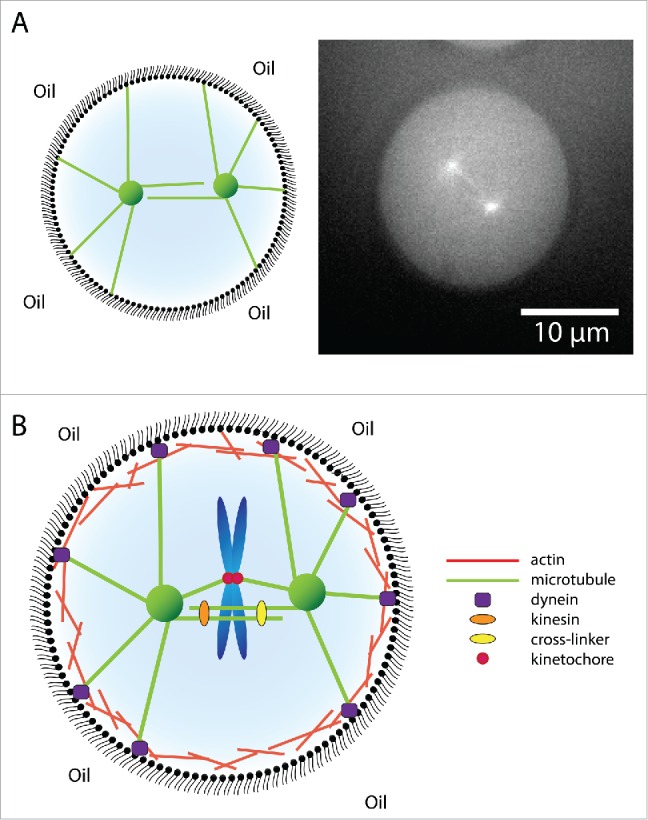
Building toward more complex *in vitro* reconstitution systems. (A) Reconstitution of mitotic spindle formation and positioning in spherical water-in-oil emulsion droplets, displaying a schematic representation (left) and immunofluorescent image showing fluorescent tubulin (right). (B) Toward creating more complex reconstitution systems in which multiple cytoskeletal components are captured into geometrical confinements together with chromosomes and additional force-generators.

Whereas most reconstitution studies have been performed using single-molecule assays, it will be a major challenge for the future to translate these studies into geometrical confinements mimicking the *in vivo* situation. The shape and nature of the confinement is an important determinant of the forces that can be generated by microtubules of the mitotic spindle.^99^ Most eukaryotic cells that lack a rigid cell wall, adopt a spherical shape when entering mitosis. Important insights will therefore come from studies on microtubule-aster positioning in spherical water-in-oil emulsion droplets generated using microfluidic technologies^124,145^ (Fig. 5A). In addition, microtubule asters growing inside micro-fabricated chambers that resemble the asymmetric shape of the bud-neck of *S. cerevisiae*, show a highly complex positioning behavior,^60^ which is at present poorly understood. In specialized cases, the mitotic spindle is positioned asymmetrically within the dividing cell, resulting in 2 daughter cells of different size and often with different cell fate. Reconstitution assays within the relevant geometrical confinements will enable the quantitative assessment of the forces involved in asymmetric spindle positioning. In addition to the shape of the geometrical confinement, the nature of the confinement can also affect microtubule dynamics and force generation. Barrier flexibility directly affects the friction between microtubules and the barrier, which is predicted to directly impact on microtubule slipping behavior.^116^ It will therefore be interesting to assess how growing microtubules respond to relatively rigid barriers compared to more flexible barriers such as emulsion droplets or the single lipid bilayer of unilamellar vesicles.

### Spindle assembly and positioning

During mitosis, the spindle is formed by the coordinated assembly of 2 microtubule asters in a bipolar orientation. It is important to note that encapsulation of 2 microtubule asters within the same geometrical confinement will give rise to additional effects compared to a single microtubule aster. In addition to the pushing forces that are generated by microtubules growing against the confinement boundary, microtubules originating from one centrosome can push against the second microtubule aster, resulting in centrosome repulsion.^29^ This force is large enough to at least partially overcome the forces generated by astral microtubules colliding with the confinement boundary and can push centrosomes to opposite sides of the confinement^60,145^ (Fig. 2D). Furthermore, the repulsion forces between 2 asters partially antagonises the centring forces generated by the pulling of cortical dynein.^145^

Mitotic spindle assembly and positioning is not only achieved by the forces generated by microtubule dynamics. Many other microtubule motor proteins and cross-linkers play important roles in mitotic spindle assembly and positioning. Plus-end directed motor-proteins of the Kinesin-5 and −12 families are for instance responsible for centrosome separation and bipolar spindle formation *in vivo*.^133^ Furthermore, passive microtubule cross-linkers of the Ase1/PRC1 family are able to generate entropic forces that increase the overlap of antiparallel overlapping microtubules.^88^ The complex process of bipolar spindle assembly in the presence of these and many other force generating complexes is at present difficult to interpret. It will therefore be important to invest in the step-wise reconstitution and quantitative assessment of mitotic spindle formation and positioning in *in vivo*-like geometrical confinements.

## General thoughts and perspectives

The technological progress in methods for purifying recombinant protein(- complexe)s have enabled extensive biochemical and biophysical characterization of numerous MAPs over the past decade. This knowledge forms a strong basis for the *in vitro* reconstitution of biological processes using synthetic biology-based approaches. Although the reconstitution of tip-tracking complexes has progressed significantly over the past years, our understanding of the role of microtubule dynamics in organelle positioning and architecture is still very limited. Also, studies on the interplay between different cytoskeletal components are only starting to unveil the first of potentially many modes of co-organization and -regulation of these components.^65^ Combining these different cytoskeletal systems into geometrical confinements to reconstitute their collective functions in various biological processes will be of great importance (Fig. 5B). To effectively reconstitute biological processes within geometrical confinements, it will be important to carefully consider the size, shape and nature of these confinements. A major challenge for future endeavors will be to increase the complexity of the described reconstituted systems toward *in vivo*-like levels.
